# Assessing the Long-term Patency and Clinical Outcomes of Venous and Arterial Grafts Used in Coronary Artery Bypass Grafting: A Meta-analysis

**DOI:** 10.7759/cureus.5670

**Published:** 2019-09-16

**Authors:** Abdul Waheed, Emily Klosterman, Joseph Lee, Ankita Mishra, Vijay Narasimha, Faiz Tuma, Faran Bokhari, Furqan Haq, Subhasis Misra

**Affiliations:** 1 Surgery, Brandon Regional Hospital, Brandon, USA; 2 General Surgery, East Tennessee State University, Johnson City, USA; 3 Surgery, HCA West Florida Consortium / Brandon Regional Hospital - USF Affiliate, Brandon, USA; 4 Surgery/vascular, Brandon Regional Hospital/hca-Usf Consortium, Brandon, USA; 5 General Surgery, Central Michigan University College of Medicine, Saginaw, USA; 6 Surgery, John H. Stroger, Jr. Hospital of Cook County, Chicago, USA; 7 Internal Medicine, Oak Hill Hospital, Tampa, USA

**Keywords:** coronary artery bypass graft (cabg), arterial grafts, venous grafts, long term outcomes, patency, mortality

## Abstract

Introduction

The long-term patency of the grafts used during the coronary artery bypass grafting (CABG) is one of the most significant predictors of the clinical outcomes. The gold standard graft used during CABG with the best long-term patency rate and the better clinical outcomes is left internal thoracic artery (LITA) grafted to the left coronary artery (LCA). The controversy lies in choosing the second-best conduit for the non-left coronary artery (NLCA) with similar patency rate as LITA. This meta-analysis examines the long-term patency and clinical outcomes of all arterial grafts versus all venous grafts used during the CABG.

Methods

A comprehensive literature search of all published randomized control trials (RCTs) assessing long-term patency and clinical outcomes of grafts used in CABG was conducted using PubMed, Cochrane Central Registry of Controlled Trials, and Google Scholar (1966-2018). Keywords searched included combinations of “CABG”, “venous grafts in CABG”, “arterial grafts in CABG”, “radial artery grafts in CABG”, “gastroepiploic artery grafts in CABG”, “patency and clinical outcomes”. Inclusion criteria included: RCTs comparing the long-term patency, and clinical outcomes of radial artery, right internal thoracic artery, gastroduodenal artery, and saphenous vein grafts used in CABG. Long-term patency of the grafts and clinical outcomes were analyzed.

Results

Eight RCTs involving 2,091 patients with 1,164 patients receiving arterial grafts and 927 patients receiving venous grafts were included. There was no difference between the long-term patency rate (relative risk (RR) = 1.050, 95% confidence interval (CI) = 0.949 to 1.162, and p = 0.344), overall mortality rate (RR = 1.095, 95% CI = 0.561 to 2.136, and p = 0.790), rate of myocardial infarction (MI) (RR = 0.860, 95% CI = 0.409 to 1.812, and P = 0.692), and re-intervention rate (RR = 0.0768, 95% CI = 0.419 to 1.406, and P = 0.392) between arterial and venous grafts.

Conclusion

The use of arterial conduits over the venous conduits has no significant superiority regarding the long-term graft patency, the rate of MI, overall mortality, and the rate of revascularization following CABG. Additional adequately powered studies are needed to further evaluate the long-term outcomes of arterial and venous grafts following the CABG.

## Introduction

Coronary artery bypass grafting (CABG) is the most efficient treatment for symptomatic multivessel coronary artery disease [1]. Every year, more than 800,000 patients undergo CABG worldwide [2]. The most effective approach used during the CABG is the anastomosis of the left internal thoracic (mammary) artery to the left anterior descending artery [1-3]. The 10-year patency rate of the left internal thoracic artery graft is 90% [4,5]. The long-term outcomes following CABG depend mostly on the patency of the vessels grafted to the coronary arteries [4,6]. Over the years, a range of arterial and venous grafts have been used during CABG to achieve maximum myocardial perfusion [2]. The great saphenous vein (GSV) is often used as an aortocoronary conduit for the non-left anterior descending (LAD) coronary artery [7,8]. Due to the larger caliber of the great saphenous vein compared to the target coronary artery, graft closure is always a possible complication. Also, the late closure is due to vein graft atherosclerosis, which results in a 50% to 60% closure rate at 10 years [8].

Recently, radial artery (RA) grafts have been revived by several studies which demonstrated excellent long-term patency [8, 9]. Many authors support the use of radial artery grafts due to the bi­ological properties, adaptation to blood flow, and minimal intimal prolifera­tion, which all lead to improved efficacy com­pared with saphenous vein grafts [6,10,11]. While several clinical studies support the routine use of radial artery instead of saphenous vein conduits, others sug­gest the opposite [8]. Controversy over the long-term patency and clinical outcomes with the use of arterial and venous grafts for non-LAD coronary arteries exists. This meta-analysis updates the previous meta-analysis (2013) by including three additional randomized control trials (RCTs) in an attempt to more precisely explain the long-term patency and clinical outcomes of all arterial and venous grafts used during CABG [8].

## Materials and methods

Study selection

**Figure 1:**
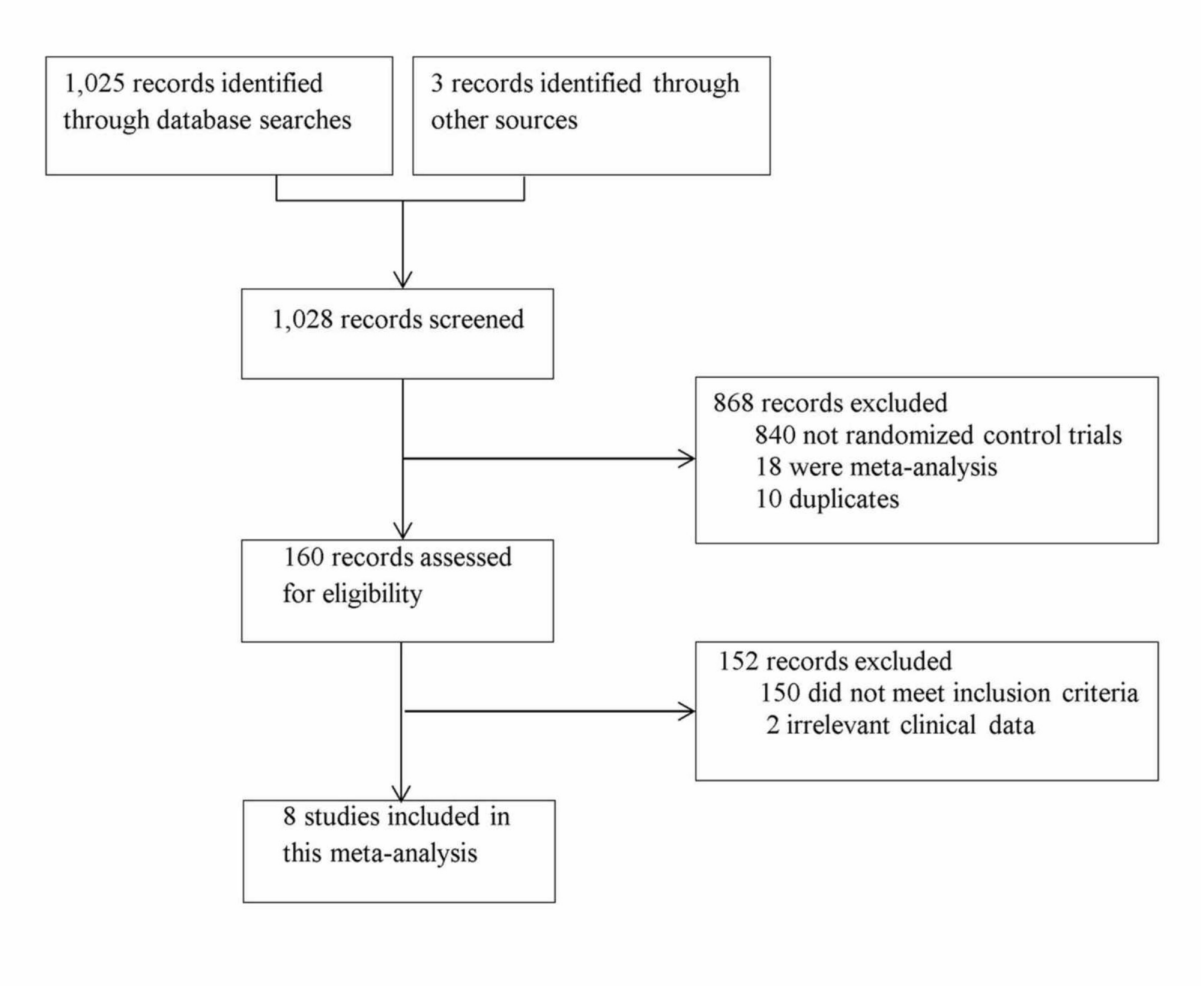
CONSORT diagram of the study selection CONSORT: Consolidated Standards of Reporting Trials

A comprehensive search of all published RCTs comparing long-term patients and clinical outcomes of the radial artery, gastroduodenal artery, and saphenous vein was conducted using PubMed, Google Scholar, and Cochrane Central Registry of Controlled Trials (1966-2018). Additional citations were searched using references retrieved from prior publications (Figure 1). The last search was conducted on November 10, 2018, and only articles conducted in English were considered. Keywords searched included combinations of ‘CABG,’ ‘venous grafts in CABG,’ ‘arterial grafts in CABG,’ and ‘patency and clinical outcomes.’ The inclusion criteria were limited to RCTs comparing the long-term patency, and clinical outcomes of the patients receiving arterial grafts (radial artery, right gastroepiploic artery, right internal thoracic artery, left internal thoracic artery) and saphenous vein grafts during CABG. In the case of duplicate publications, only the most recent and updated report of the clinical trial was included.

Data extraction

Articles retrieved from this search were assessed for eligibility and data pertaining to patients, intervention, comparison groups, outcomes, and methodology were abstracted. The primary clinical outcome of interest was long-term patency. Secondary outcomes included were myocardial infarction, overall mortality, and revascularization (Table 1).

**Table 1:** Characteristics of included studies PMID: PubMed IDentifier; RA: Radial artery; SVG: Saphenous vein graft; SV: Saphenous vein; DM: Diabetes mellitus; LITA: Left internal thoracic artery; RITA: Right internal thoracic artery; LAD: Left anterior descending; CABG: Coronary artery bypass grafting.

Study (Author, Year) PMID#	Country	Main inclusion/exclusion criteria (ex: age of patients)	Number of patients (#receiving arterial/# receiving venous)	Details pertaining to grafts (arterial and venous grafts)	Follow-up time	On-pump vs. off-pump	Outcomes analyzed
Petrovic et al., 2015 [3] PMID. 26466996	Serbia	Inclusion criteria: One target vessel disease for RA/SVG graft, had at least 80% stenosis, was at least 1.5 mm in diameter, had no diffuse distal disease. Exclusion criteria: Positive Allen’s test, history of Raynaud's syndrome or vasculitis, single-vessel disease, <80% stenosis, patient undergone any concomitant acquired or congenital cardiac or aortic surgery.	Total patients = 200; patients with RA graft = 100, patients with SV graft = 100	RA grafts were placed either on first (50%) or on second (15%) obtuse marginal branch. RA grafts were never placed to the right coronary artery or diagonal branch if they were previously occluded.	8 years	100% on-pump	Late graft patency: RA = 92%, SVG = 86%; Mortality: RA = 12 patients, SVG = 12 patients; Myocardial infarction: RA = 7 patients, SVG = 7 patients; Repeat myocardial revascularization: RA = 10 patients, SVG = 16 patients
Zhu et al., 2014 [5] PMID. 23671205	Australia	Inclusion criteria: This study included all patients who had undergone at least one elective, protocol- or symptom-directed angiogram or CTA and at least one postoperative lipid assay. Exclusion criteria: Patients with no pre-operative lipid profile.	Total patients = 413, SV grafts = 311, LITA grafts = 408, RA grafts = 226, RITA grafts = 137		9.4 years	100% on-pump	Graft failure: SV graft failure = 88 (20%). All arterial graft failure rate = 66 (8.6%)
Gaudino et al., 2005 [9] PMID. 16159829	Italy	Inclusion criteria: Primary elective isolated CABG, previous percutaneous coronary angioplasty with successful stent implantation in any coronary vessel 1.2 mm in diameter at least one month before surgery with preoperative angiographic demonstration of patency (N = 60, control group), intracoronary stent, angiographic evidence of triple-vessel coronary artery disease with a disease (i.e., proximal stenosis 70%) graftable (i.e.,1 mm in diameter) obtuse marginal artery (OM) type I according to the classification proposed by McAlpine, good preoperative left ventricular function (ejection fraction 0.50) and no preoperative evidence or history of lateral or posterolateral myocardial infarction. Exclusion criteria: Patients who underwent stent implantation one month before surgery.	Total patients = 120, RA grafts = 40, SV grafts = 40, RIMA grafts = 40; Total patients followed by angiography = 120, RA grafts followed by angiography = 40, SV grafts followed by angiography = 40, RIMA grafts followed by angiography = 40	RA, SV, and RIMA were grafted to the circumflex coronary artery.	5.4 years	100% on-pump	Late graft patency artery = 73/80, Veins = 25/40
Dreifaldt et al., 2013 [12] PMID. 23684156	Sweden	Inclusion criteria: Patients who had at least three-vessel coronary artery disease. Exclusion criteria: Age > 65 years, left ventricular ejection fraction 120 mmol/L, use of anticoagulants, coagulopathy, allergy to contrast medium, positive Allen’s test result or an abnormal result of Doppler study of the arms, a history of vasculitis or Raynaud’s syndrome, bilateral varicose veins, or previous vein stripping.	Total patients in the study = 108; Total patients with angiographic follow-up = 99; RA grafts = 108; SV grafts = 108	Each patient received one LITA, one RA, and one No Touch (NT) SV graft as conduit material. The LITA was used to bypass the left anterior descending coronary artery, and the RA and NT SV grafts were randomized to bypass either the left or the right coronary territory.	3 year	100% on-pump	Graft patency: RA graft patency = 81patients, SV graft patency = 93 patients. Cardiac death: RA = - ; SV = - . Myocardial infarction: RA = - ; SV = - . Repeat coronary intervention: RA = - ; SV = - .
Deb et al., 2012 [13] PMID. 22742399	Canada	Inclusion criteria: Age < 80 years, three-vessel disease, non-LAD. Exclusion criteria: Positive Allen’s test, Vasculitis or Raynaud’s syndrome, bilateral varicose veins.	Total patients in the study = 510; RA graft = 510; SV graft = 510; Number of patients with angiographic follow-up = 269; RA grafts in follow-up patients = 269; SV grafts in follow-up patients = 269	RA grafted to the right coronary artery or left circumflex artery. SV grafted to the opposite territory.	7.7 years	-	Graft failure: RA = 28/269; SV = 50/269. Cardiac death: RA = 0/510; SV = 1/510. Myocardial infarction: RA = 2/510; SV = 3/510. Repeat coronary intervention: RA = 3/510; SV = 12/510
Deb et al., 2014 [14] PMID. 25109754	Canada	Inclusion criteria: Age < 80 years, patients with triple-vessel disease. Exclusion criteria: Contraindication for the use of the RA (i.e., positive Allen’s test), abnormal arterial upper limb duplex scan, a history of vasculitis (Raynaud’s syndrome) or the SV (i.e., bilateral varicosities or vein stripping). Further exclusion criteria were factors limiting follow-up research angiography, which included creatinine greater than 180 mmol/L, severe peripheral vascular disease limiting femoral access, coagulopathy or obligatory use of anticoagulants, known allergy to radiographic contrast, pregnancy, and geographic inaccessibility.	Total patients = 529; patients for long-term angiographic follow-up = 269; Total DM patients = 148; Total DM patients for long-term angiographic follow-up = 83; Total non-DM patients with long-term angiographic follow-up = 186; Total DM patients receiving RA grafts = 83; Total non-DM patients receiving RA grafts = 186; Total DM patients receiving SV grafts = 83; Total non-DM patients receiving SV grafts = 186	The RA was randomized to the inferior (right coronary artery) or lateral (circumflex artery) region of the heart. The SV graft was placed at the opposing territory (circumflex artery or right coronary artery).	After 5 years	100% on-pump	Late graft patency: DM and RA graft = 91.5%; DM and SV graft = 79.7%; Non-DM with RA graft = 90.3%; Non-DM with SV graft = 86.2%. Complete graft occlusion: DM and RA graft = 4.8%; DM and SV graft = 25.3%; Non-DM with RA graft = 10.8%; Non-DM with SV graft = 15.6%
Hayward et al., Group 1, 2011 [15] PMID. 21392707	Australia	Inclusion criteria: Age > 70 years, three-vessel disease, non-LAD. Exclusion criteria: Positive Allen’s test, vasculitis or Raynaud’s syndrome, bilateral varicose veins.	Total patients in study = 365. Total patients for angiographic follow-up = 227, RA grafts = 186, SV grafts = 0, RIMA grafts = 179, RA drafts for follow-up = 122, RIMA drafts for follow-up = 105	The largest non-LAD target was randomized to receive either RA or RIMA.	5.5 years	100% on-pump	Graft failure: RA = 13/122, RIMA = 12/105
Hayward et al., Group 2, 2011 [15] PMID. 21392707	Australia	Inclusion criteria: Age > 70 years, three-vessel disease, Non-LAD. Exclusion criteria: Positive Allen’s test, Vasculitis or Raynaud’s syndrome, bilateral varicose veins.	Total patients in study = 214, total patients for angiographic follow-up = 110, RA grafts = 104, SV grafts = 110, RIMA grafts = 0, RA drafts for follow-up = 51, SV drafts for follow-up = 59	The largest non-LAD target was randomized to receive either RA or SV graft.	5.5 years	100% on-pump	Graft failure: RA = 4/51, SV = 9/59. Cardiac death: RA = 4/113, SV = 2/112. Myocardial infarction: RA = 4/113, SV = 4/112, Repeat coronary intervention: RA = 1/113, SV = 4/112
Collins et al., 2008 [16] PMID. 18506009	UK	Inclusion criteria: Age 40-70 years, two-vessel disease, and left circumflex coronary artery stenosis. Exclusion criteria: LV ejection fraction <25%, positive Allen’s test, a history of Raynaud’s syndrome, bilateral varicose veins.	Total patients in the study = 142, patients with RA graft = 82, with SV graft = 60. Patients with angiographic follow-up = 103, RA grafts for angiographic follow-up = 59, SV grafts for angiographic follow-up = 44	RA or SV grafted to left circumflex coronary artery	5 years	100% on-pump	Graft patency: RA = 98.3%, SV = 86.4%

Statistical analysis

For each trial, relative risk (RR) with a 95% confidence interval (CI) for long-term patency, overall mortality, myocardial infarction, and revascularization were calculated. The standard difference in mean (SDM) with 95% CI were calculated for patency rate, myocardial infarction graft failure, and revascularization. A meta-analysis of the pooled data was performed using the Comparative Meta-Analysis software Version 3 (Biostat, Englewood, NJ). For individual studies reporting zero events in any group, a continuity correction factor of 0.5 was adopted to calculate the RR and variance. Both the fixed effects model and the random-effects model were considered, depending on the heterogeneity of the included studies. To assess the heterogeneity between studies, both Cochrane’s Q statistic and I2 statistic were used. Heterogeneity was considered statistically significant when p < 0.05 or I2 > 50. If heterogeneity was observed, data were analyzed using a random-effects model. In the absence of heterogeneity, a fixed-effects model was assumed.

For all the outcomes, publication bias was first evaluated using a funnel plot and further evaluated with Egger’s and Begg’s tests. A two-tailed p-value of <0.05 was considered statistically significant.

## Results

Demographic characteristics of the studies

A total of eight RCTs were identified, involved 2,091 patients, of which 1,164 patients received arterial grafts and 927 patients received venous grafts. The primary outcome was long-term graft patency. Mortality, re-intervention, and rate of myocardial infarction were identified as secondary outcomes from these studies** **(Table 1).

Assessment of the long-term graft patency

**Figure 2:**
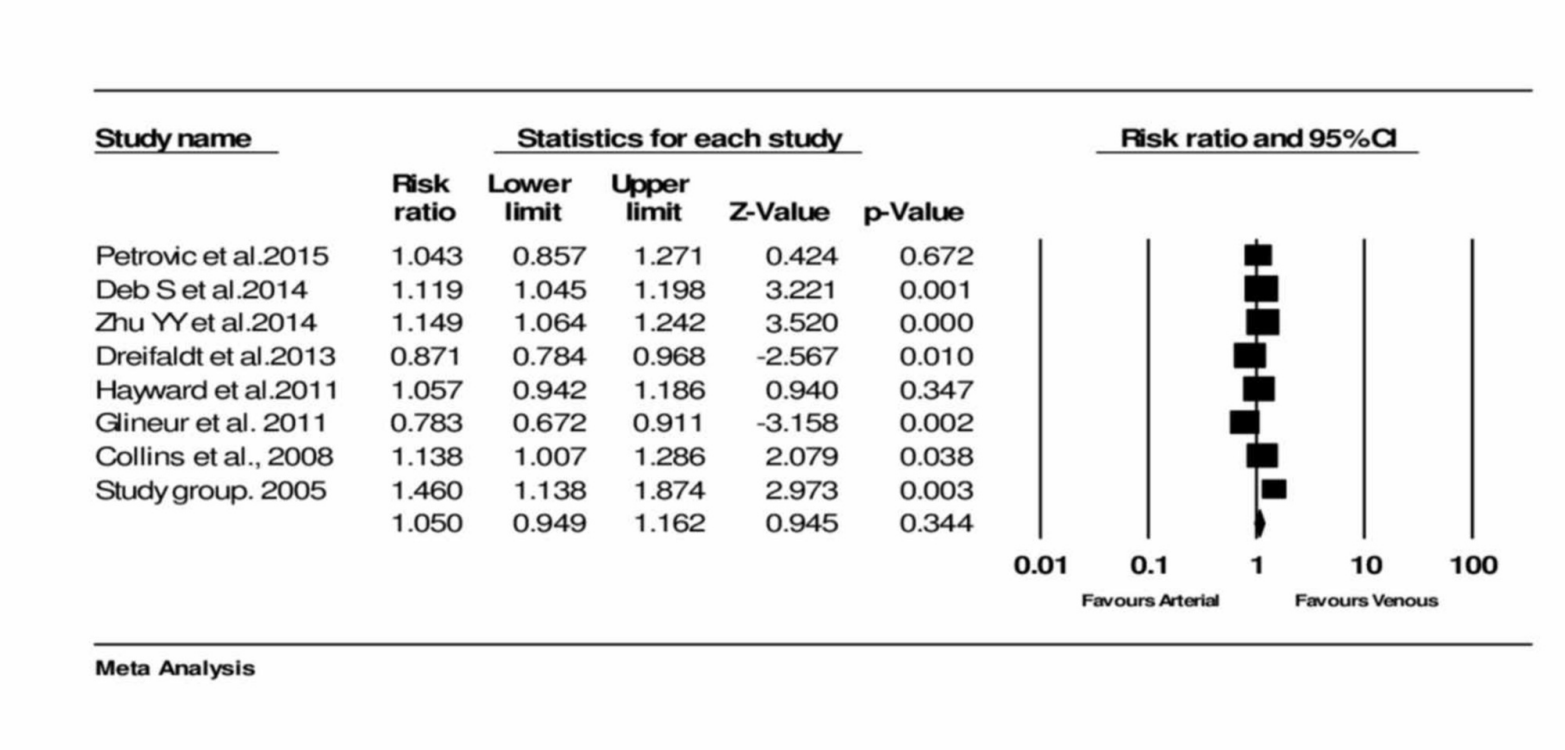
Forest plot: The long-term patency of arterial and venous conduits Petrovic et al., 2015 [3] Deb et al., 2014 [14] Zhu et al., 2014 [5] Dreifaldt et al., 2013 [12] Hayward et al., 2011 [15] Collins et al., 2008 [16]

All eight trials reported on the long-term patency of the arterial and venous grafts. No significant heterogeneity was noticed amongst the trials. Analysis resulted in no significant difference in long-term patency between the two groups (RR = 1.050, 95% CI = 0.949 to 1.162, and p = 0.344)** **(Figure 2) (Table 1).

Assessment of the overall mortality

**Figure 3:**
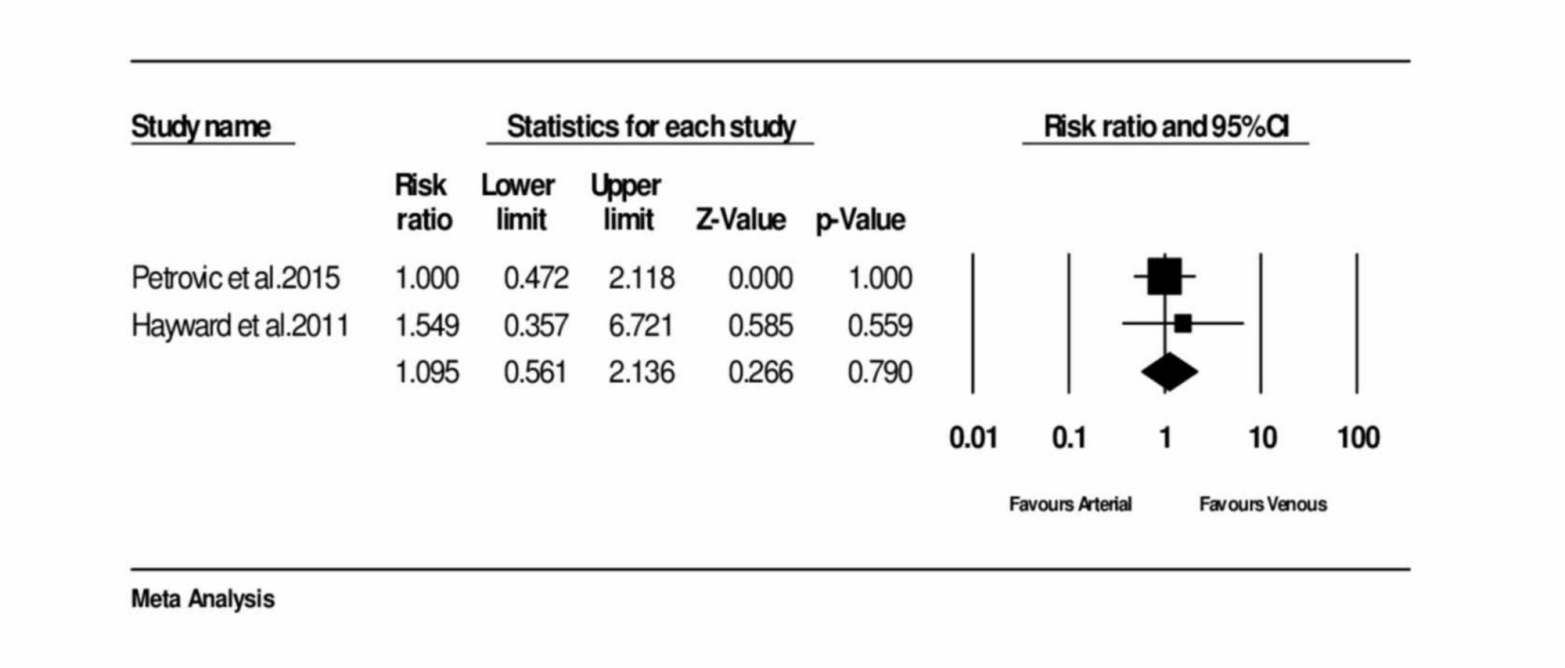
Forest plot: Overall mortality of arterial and venous conduits Petrovic et al., 2015 [3] Hayward and Buxton, 2011 [15]

Two studies provided the data on overall mortality [3,12]. No significant heterogeneity was noticed between the trials. The analysis resulted in no significant difference in the overall mortality rate between the two groups. The pooled RR was 1.095 (95% CI = 0.561 to 2.136, and p = 0.790) (Figure 3) (Table 1).

Assessment of the incidence of myocardial infarction

**Figure 4:**
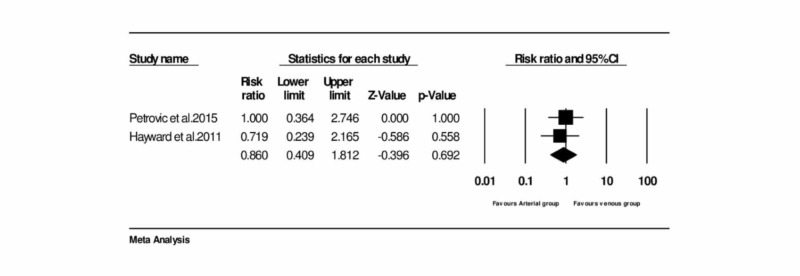
Forest plot: Incidence of myocardial infarction between arterial and venous conduits Petrovic et al., 2015 [3] Hayward and Buxton, 2011 [15]

Two trials provided the data on overall mortality [4, 8]. No significant heterogeneity was noticed between the trials. This current meta-analysis reports no significant difference in the rate of myocardial infarction between the two groups. The pooled RR was 0.860 (95% CI = 0.409 to 1.812, and P = 0.692) (Figure 4) (Table 1).

Assessment of the re-intervention rate

**Figure 5:**
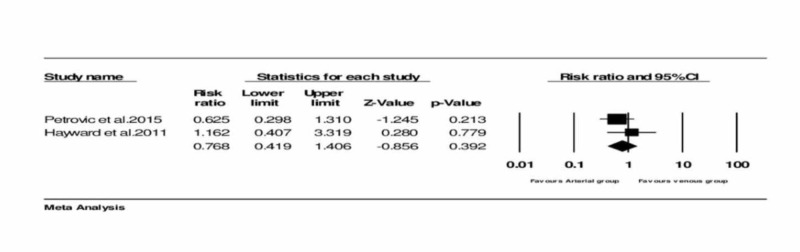
Forest plot: Re-intervention rate between arterial and venous conduits Petrovic et al., 2015 [3] Hayward and Buxton, 2011 [15]

Two trials provided the data on the re-intervention rate [3, 12]. No significant heterogeneity was noticed between the trials. The analysis resulted in no significant difference in the re-intervention rate between the two groups. The pooled RR was 0.0768 (95% CI = 0.419 to 1.406, and P = 0.392) (Figure 5) (Table 1).

Publication bias

**Figure 6:**
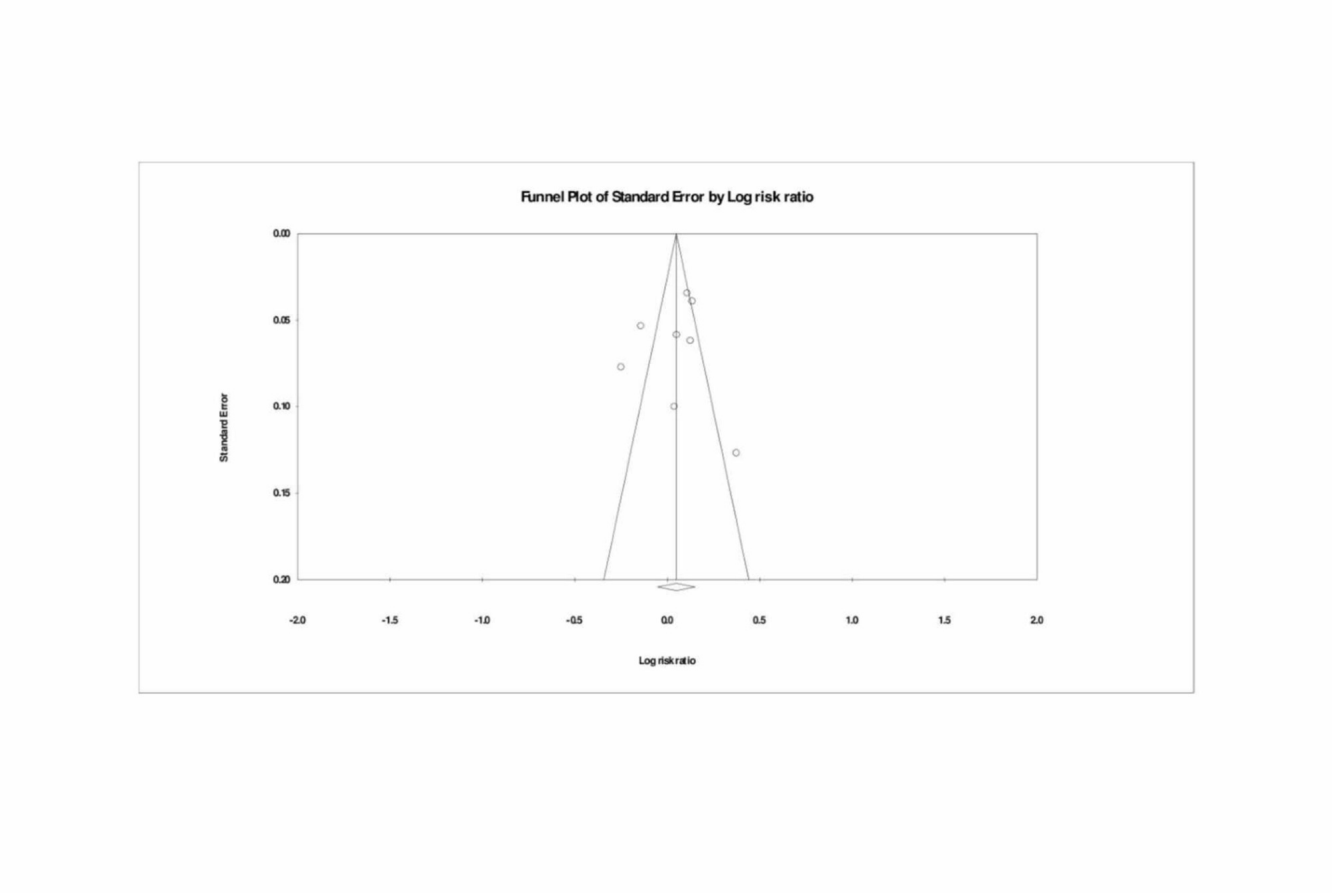
Funnel plot assessing publication bias (analyzing the long-term patency of the arterial and venous grafts)

A funnel plot was used to assess for publication bias visually, and both Egger’s and Begg’s tests were performed to calculate publication bias. There was no obvious evidence of asymmetry on the funnel plot (Figure 6). Furthermore, there was no evidence of the publication bias for the primary endpoint of this study (long-term patency of arterial and venous grafts in CABG) by either the Egger’s (p = 0.671) or Beggs’s test (p = 0.901).

## Discussion

Coronary-artery bypass grafting (CABG) was first introduced in the 1960s, which became the standard of care for symptomatic patients with coronary artery disease [1]. The long-term patency of the grafts used during CABG is one of the most critical determinants of the excellent clinical outcomes [4, 13]. The choice of conduit used during the CABG with the best long-term outcomes has evolved over the years [17]. In 1964, Kolesov performed the first successful internal mammary artery-coronary artery anastomosis [18, 19]. During the same decade, Favaloro reported using saphenous vein to restore coronary artery blood flow [20].

In 1973, Carpentier et al. used radial artery for the first time as a conduit for the CABG, but this idea did not gain popularity due to the early rejection of the graft [21, 22]. The idea of using radial artery as an effective graft reemerged in 1990 when early graft rejection of radial artery was prevented with the use of calcium channel blockers [13, 22-24].

The patency of grafts has primarily assessed the success rate of coronary artery bypass grafting [14, 25]. Unfortunately, there is no clear evidence for selecting best second graft with comparable long-term patency to LITA-to-LAD graft. Several RCTs have reported discordant results on the long-term patency superiority of a second arterial or venous conduit [4, 15]. This study compares the long-term patency of all the arterial and venous grafts and associated long-term clinical outcomes.

The patency of vessels grafted to the coronary arteries has been divided into three stag­es: early patency (<6 months), medium-term patency (6-36 months), long-term patency (>36 months) [8]. Several factors contribute to the patency of a grafted vessel, and one of them is the biological properties of the vessel wall [2, 8]. There is a general agreement that long-term patency of arteries and veins are dependent on their biological makeup and their resistance to atherosclerosis [2, 8, 26]. The arterial conduits are considered more favorable for the high-pressure arterial environment in the coronary arteries [8]. The shear stress in the arterial environment induces compensatory mechanisms in endothelial cells of the arterial walls that lead to the release of local vasodilators like nitric oxide and prostaglandins and also inhibit the constricting factors like endothelian [24, 27]. All these biochemical changes enable the arterial wall to be more resistant to the high arterial pressure [8]. However, merely the quality of the evidence above does not lead to the definitive conclusion to support the use only arterial grafts for CABG [8].

There has been marked variability in the reported long-term patency and graft occlusion of arterial and venous grafts. Athanasiou et al. included both randomized trials and observational studies in a meta-analysis to compare the patency rates at follow-up intervals of >5 years. They concluded that rate of late graft occlusion was significantly reduced in radial artery group compared to saphenous venous group [for observational and randomized trials, OR = 0.520 (95% CI: 0.34 to 0.79, p = 0.002); for RCTs alone, OR = 0.49 (95% CI = 0.31 to 0.77, p = 0.002)] [2].

Another single-center study, Radial Artery Versus Saphenous Vein Patency (RSVP), reported that complete graft occlusion at a follow-up of 5.5 years was markedly less frequent in radial grafts compared to the saphenous vein group [16]. On the other hand, the RCT conducted by Buxton et al., comparing the RA with the free right internal thoracic artery (RITA) and the saphenous vein graft (SVG), did not support the superior patency of the RA compared with the RITA or the SVG [28]. Similar findings were reported in a recent RCT conducted by Petrovic et al. [3]. They enrolled 200 patients and randomly assigned them to the radial artery group (100 patients) and saphenous venous group (100 patients). At a follow-up of eight years, the patency rate was 92% in the radial artery group and 86% in the saphenous venous group (p = 0.67) [3]. These results are consistent with the findings of this meta-analysis.

For the secondary outcomes, this me­ta-analysis suggests that the overall mortality rate, revascularization, and rate of myocardial infarction are not significantly different in either arterial or venous groups. In the Stand-in-Y trial, the survival rate was similar in patients who received a radial artery compared with a second ITA graft. These results are also consistent with the findings of this meta-analysis. They enrolled 3,102 patients; 1,554 were randomly assigned to receive single internal-thoracic-artery grafts, and 1,548 received bilateral internal-thoracic-artery grafts. At five years of follow-up, the mortality rate was 8.7% in those patients who received bilateral-grafts and 8.4% in those who received single artery grafts (Hazard ratio (HR) = 1.04; 95% CI = 0.81-1.32; P = 0.77), and the mortality rate of death from myocardial infarction was 12.2% (HR = 0.96; 95% CI = 0.79-1.17; P = 0.69). On the other hand, in a single-center observational study, the survival rate was enhanced with the use of the radial artery compared with a saphenous vein [29]. In 2004, Zacharias et al. reported the six-year clinical outcomes of propensity-matched patients undergoing LIMA-LAD, using either RA or SVG for additional graphs as a secondary conduit. In 925 patients, they found cumulative survival was better with the RA grafts [30].

Some of the limitations should be acknowledged about the present study, and the results should be interpreted with caution. First of all, all the studies included in this meta-analysis are small, with the largest having 529 participants. Also, studies do not have a fixed follow-up interval. Other limitations of this study include the power of individual secondary outcomes.

## Conclusions

Despite these limitations, the results from this meta-analysis indicate that the use of arterial conduits over the venous conduits for CABG has no statistically significant effect regarding long-term graft patency, the rate of MI, overall mortality, and the rate of revascularization following CABG. More massive, multi-center randomized control trials are needed to be done in order to determine long-term patency of arterial and venous grafts. It is recommended that the studies must take into consideration the effects of various drugs the patients are taking and their influence on the long-term patency and clinical outcomes.
